# Anesthetic-Induced Developmental Neurotoxicity: Cognitive Sequelae of Early Exposure

**Published:** 2026-03-20

**Authors:** David Parvizi, Sugeeth Kandikattu, Gregory Ayzenberg, Manas Aavula, Mohammad Khaleefa, Devendra K Agrawal

**Affiliations:** 1 Department of Translational Research, College of Osteopathic Medicine of the Pacific, Western University of Health Sciences, Pomona, California 91766, USA; 2 American University of Antigua School of Medicine, New York, NY 10006, USA.

**Keywords:** Anesthesia, Apoptosis, Dexmedetomidine, Epigenetic dysregulation, Executive function, GABA agonists, Neurodevelopment, Neurotoxicity, N-methyl-D-aspartate (NMDA), NMDA receptor antagonism, Pediatric anesthesia, Synaptogenesis

## Abstract

Preclinical studies showing widespread neuronal apoptosis and long-term behavioral deficits when exposed to common anesthetics raised concerns for anesthetic-induced neurotoxicity in pediatric patients during periods of rapid synaptogenesis. N-methyl-D-aspartate receptor antagonists have been shown in animal studies to disrupt activity-dependent plasticity, hinder mitochondrial function, and induce epigenetic dysfunction. These studies have led to regulatory warnings and investigation into the potential long-term cognitive and neurodevelopmental consequences in children who have been exposed to anesthesia before the age of three. Human data, including cohort studies and randomized controlled trials, such as the General Anesthesia versus Spinal Anesthesia trial and the Pediatric Anesthesia Neurodevelopmental Assessment studies have found that a single, brief, exposure to anesthesia in infancy is not associated with any measurable deficits in intelligence. However, multiple, prolonged exposures can be associated with dysfunctions in learning, behavior, and social communication but remains a question due to confounding variables across studies. The current literature does not support the delay of medically necessary procedures in pediatric populations due to potential anesthetic toxicity. Continuing research aims to delineate mechanisms of injury, identify vulnerable populations, and develop mitigation strategies such as alternative agents and neuroprotective adjuvants.

## Introduction

The emergence of neurotoxicity concerns with anesthesia exposures in pediatric populations emerged from animal studies in the late 1990s and early 2000s which demonstrated neuronal cell death and behavioral deficits after common anesthetic agent exposures in developing brains [1-10]. A single, brief anesthesia exposure in young children has been shown not to cause any detectable neurodevelopmental deficit, however, there is concern regarding prolonged exposure or multiple exposure in pediatric patients under the age of three years old [11]. Around 20% of the 20 million U.S. children below the age of five years old undergo general anesthesia or deep sedation each year, making this a significant public health consideration [12]. NMDA antagonists such as ketamine, isoflurane, and nitrous oxide as well as GABA agonists including volatile anesthetics, midazolam, and propofol have shown dose-dependent and age-dependent neuronal cell death with deficits in learning, memory, and behavior in rodent studies and non-human primate studies. These results prompted the FDA to issue a Drug Safety Warning in 2016 regarding prolonged or repeated anesthetic use in children under the age of three [13].

Although, there is no significant human clinical evidence that supports these animal findings. The General Anesthesia versus Spinal Anesthesia (GAS) trial and the Pediatric Anesthesia Neurodevelopmental Assessment (PANDA) trial showed no negative effects on IQ or general intelligence from a single brief anesthetic exposure in infancy [14]. Several studies have confirmed no association with neurodevelopmental impairments in later childhood with anesthesia exposure less than one hour. These studies provide relevant results since 50% of anesthetic procedures in young U.S. children are one hour or less [15]. However, multiple exposures or prolonged exposures can carry a greater risk for children. Retrospective studies have shown an association between multiple anesthetic exposures and behavioral deficits including executive function, social communication, and ADHD diagnosis. On the contrary, The Mayo Anesthesia Safety in Kids study examined multiple anesthetics which would not demonstrate differences in general intelligence as the primary outcome [16].

Broad measures of general intelligence seem to be unaffected whereas subtle differences in specific neurodevelopmental domains can occur. Meta-analyses suggest potential impacts on behavioral regulation, executive function, academic achievement and school readiness, and learning disabilities [17]. In terms of clinical implications, the current literature does not support delaying medically necessary procedures to avoid anesthesia in young children. Factors such as biological, environmental, and social carry far more important weight than anesthetic exposure for neurodevelopmental outcomes in healthy children. The underlying pathology needed to be fixed via anesthesia are important to consider when assessing neurodevelopmental risk [18]. Alternatives such as regional anesthetic techniques with adjuvants can have shown to potentially mitigate neurotoxicity risk and could be considered for smaller procedures.

The clinical and public health significance of anesthetic toxicity concerns is significant, as the scale of exposure is increasing each year but also reassuring at the same time based on the current evidence of safety for a single, brief anesthetic episode remains quite safe [19]. Multiple, or prolonged exposures, remain to be an area of concern, as there is not enough literature covering the outcomes. The possibility that behavioral deficits represent a phenotype of injury remains a source of heated debate amongst clinical anesthesiologists, as the findings are more consistently reported in humans and have been shown in findings from nonhuman primates [20]. Although the FDA 2016 Drug Safety Warning has provided compelling preclinical evidence on the safety of neurotoxicity of anesthesia in pediatric populations, it has led to uncertainty that must be balanced against the risks of delaying necessary care. The challenge that anesthesiologists face is in translating animal model findings to human populations with more complex development.

The aim of this review is to evaluate the current evidence on neurotoxicity in pediatric populations with early-life exposure with an emphasis on potential long-term cognitive and neurodevelopmental consequences. This review also seeks to examine proposed mechanisms of neuronal injury, identify gaps in knowledge, and explore future directions for safer means of anesthesia exposure in pediatric patients.

## Neurodevelopmental Vulnerability and Mechanistic Basis

The developing brain exhibits significantly more vulnerability to anesthetic agents during times of increased synaptogenesis which generally occurs within the third trimester of gestation and continues on until about the first three years of life [21]. During this time, a critical window period occurs in which the brain undergoes rapid proliferation, migration, differentiation, synaptogenesis, myelination, and programmed apoptosis. This process is profoundly vulnerable to any type of environmental disruption [22].

**Figure 1:**
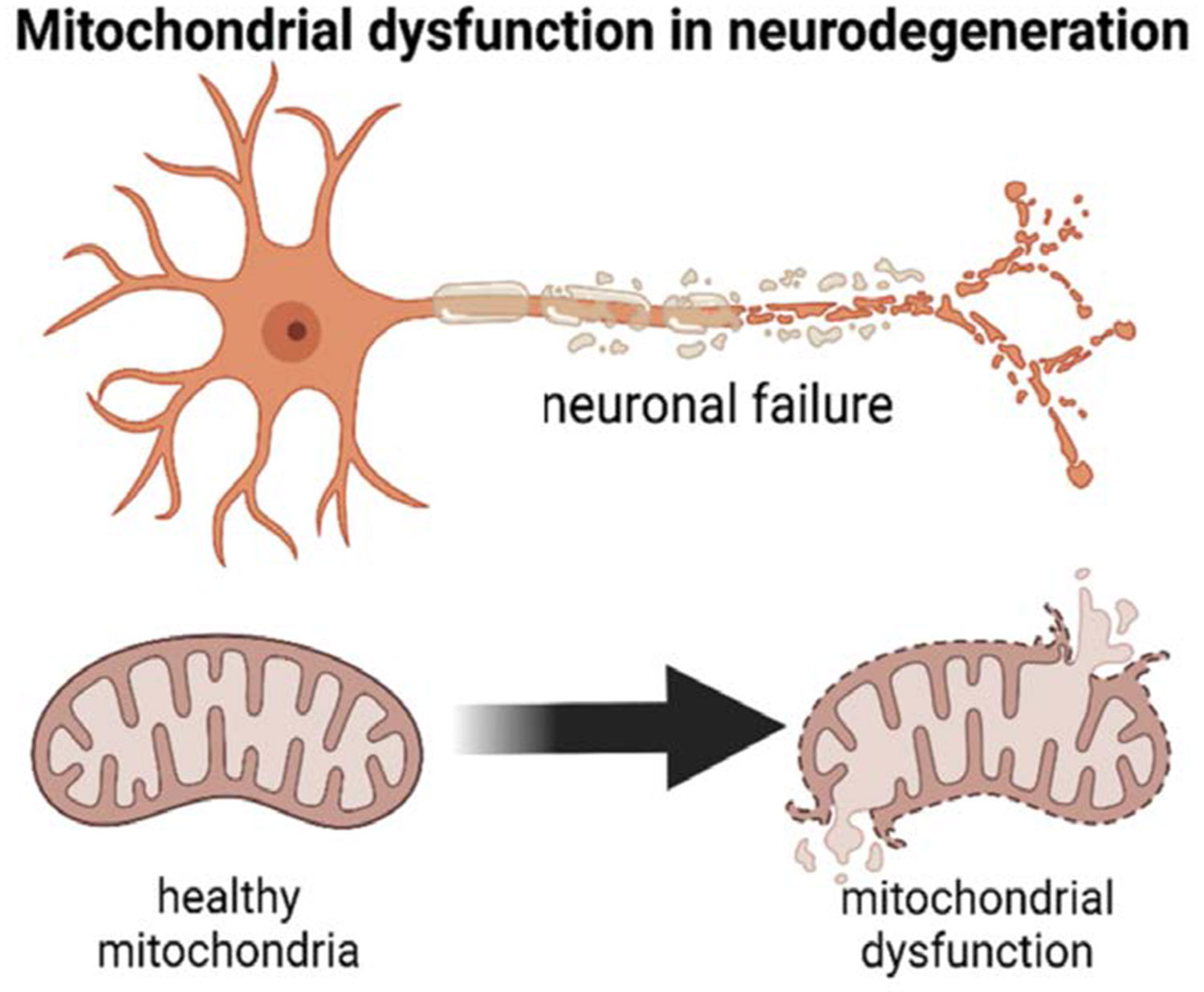
This schematic diagram summarizes the proposed cellular and molecular mechanisms of anesthetic-induced neurotoxicity during early-life synaptogenesis. Anesthetic agents act through NMDA receptor antagonism and GABA receptor potentiation, reducing neuronal activity during a critical developmental window. Downstream effects include caspase-3–mediated apoptosis, mitochondrial dysfunction, oxidative stress, impaired synaptogenesis, disrupted dendritic architecture, and epigenetic alterations, ultimately contributing to altered neural circuit development and potential long-term neurodevelopmental consequences.

Anesthetic-induced neurotoxicity disrupts two large neurotransmitter systems which include the inhibition of NMDA (N-methyl-D-aspartate) receptors and the activation of GABA (γ-aminobutyric acid) receptors. These two mechanisms will decrease neuronal activity during a time when activity-dependent plasticity is integral for normal brain development [23]. This transient inhibition triggers several pathological cascades. Anesthetics can generate accelerated apoptotic neurodegeneration in developing neurons, which has been cited in the literature across primates and rodents in previous studies [24]. In addition to neuronal cell death, the remaining neurons experience chronic neuropathology which includes unusual dendritic morphology, dampened synaptogenesis, and disrupted synaptic plasticity (Figure 1).

Recent studies have revealed that anesthetics can cause mitochondrial dysfunction and dysregulate epigenetic modifications as well [25]. Recent studies have shown that exposure to propofol decreases ATP production and disrupts expression of synaptic markers such as PSD95 and c-Fos as well as disrupting histone acetylation and DNA methylation patterns that have large impacts on DNA transcription [26]. These subtle changes could explain why brief exposure to anesthesia could have persistent effects and potentially even intergenerational transmission [27]. The vulnerability to anesthesia depends on dose and age. In animal models, neurotoxicity was found to develop only after being exposed above threshold doses and durations during the critical neurodevelopmental window of maximal synaptogenesis. Exposure outside of this window or below the anesthetic threshold doses did not produce any apparent neurotoxicity [28]. Regarding human development, the brain matures from gestation to adolescence, with different parts of the brain maturing at different rates. The period from term birth to about two years is signified by rapid and dynamic brain development, with significant implications for cognitive development and risk of neuropsychiatric disorders [29].

Different regions of the brain and brain behavioral domains experience different sensitivities based on their developmental trajectories. Primary sensory cortices develop earlier than their associated cortices, suggesting a hierarchical pattern of critical periods across the cortex. This is significant in that the timing of anesthetic exposure could likely determine which neural circuits, and psychological processes will be affected the most. The full mix of biological and environmental influences the brain encounters, otherwise known as the concept of the dynamic neural exposome, emphasizes that anesthetic effects occur within a complex developmental context. Several factors including the surgical pathology, comorbidities, perioperative stress, and socioeconomic status interact with anesthetic exposure and have influence over outcomes [30].

The cellular and molecular mechanisms of anesthetic-induced injury involve several connected pathways that disrupt critical neurodevelopmental processes during times of heightened synaptogenesis. These mechanisms operate through both acute cellular damage and chronic dysregulation of developmental programs (Figure 1).

Anesthetics generally act by inhibiting NMDA receptors and activating GABA receptors, both of which decrease neuronal activity during a critical period when activity-dependent plasticity is essential for normal brain development. The temporary inhibition activates accelerated apoptotic neurodegeneration in developing neurons across several different species [31]. During development, GABA transitions from an excitatory neurotransmitter to an inhibitory neurotransmitter, and anesthetic interference with GABAergic and glutamatergic systems during this period has been shown to be damaging [32].

As mentioned earlier, propofol can cause significant mitochondrial dysfunction, reduce ATP production and disrupt cellular energy metabolism. Studies that used human cerebral organoids showed that propofol exposure decreases ATP levels and reduces expression of CKMT1B, an enzyme crucial for energy metabolism [33]. This mitochondrial impairment contributes to both acute cell death and chronic cellular dysfunction in surviving neurons.

## Evidence from Experimental and Clinical Studies

Studies done on animals have shown consistently that an early-life exposure to general anesthetics causes acute neuronal and long-term cognitive deficits. The effects are largely dependent on the developmental timeframe, duration of exposure, and the specific type of anesthetic agent used. Volatile therapies such as sevoflurane and isoflurane as well as GABA agonists and NMDA antagonists that are used during critical brain development periods induce an apoptotic cell death on a large scale in rodents and primates [34]. As a result, neurogenesis, glial death, and abnormal axon formation ensues. The response to these neurotoxins is dependent on age and brain region, with the period of intense synaptogenesis being the most vulnerable time [35].

The long-term consequences are still being assessed; however, studies have shown that animals exposed to anesthetics during early postnatal periods demonstrate persistent deficits in learning and memory that extend into early adulthood. One study showed that continued isoflurane exposure in rodents induced decreases in activity levels, motor coordination, socialization, and increased anxiety-related behaviors [36]. This study also noted that repeated exposures cause more harm than a single exposure. In the experiment, sevoflurane that was given for 2 hours daily for 3 days caused diminished cognitive outcomes compared to mice that were exposed once in three days. The main mechanism by which these deficits occur is thought to occur due to disrupted growth and signaling, reactive oxygen-species mediated stress, and direct neuronal modulation [37]. Recent literature suggests that anesthetics cause persistent epigenetic dysfunction such as altered chromatin accessibility and DNA methylation, leading to impaired hippocampal neurogenesis. Moreover, sevoflurane given in the neonatal period created lasting changes in dendritic morphology, increased dendritic complexity, and improper regulation of genes responsible for synaptic plasticity [38].

These animal models have shown significant results; however, the clinical importance remains to be a question. Many of the rodent studies administered anesthetics at supraclinical doses and durations and failed to maintain physiological homeostasis during anesthesia. Evaluation of neonatal mice exposed to isoflurane caused severe respiratory and metabolic derangements which may not correlate clinically in humans [34].

The translational strengths, however, have been invaluable in identifying the exact pathology of neurotoxic mechanisms that general anesthetics can induce. Neuronal apoptosis, impaired neurogenesis, and altered synaptic development have all been shown consistently throughout the literature in animal studies. This creates a high possibility that these effects could occur in humans as well [39]. Non-human primate studies are relevant given the similar developmental trajectory to humans. These models have shown similar morphologic changes and behavioral deficits that were found in rodent studies [40]. Recent insights from animal studies advances the mechanistic understanding of persistent dysregulation following early anesthetic exposure. One study showed that neonatal midazolam exposure in mice created lasting neurological and cognitive deficits in mice but were reversible with voluntary exercise [41]. This study showed the use of possible therapeutic targets which can potentially reverse neurotoxicity caused by early anesthetic exposure.

The only human randomized controlled trial represents the strongest human evidence in terms of anesthetic toxicity and neurocognitive dysfunction. This international, multicenter study saw no significant difference in neurodevelopmental outcomes at five years of age between infants that were randomized to general anesthesia versus regional anesthesia for hernia surgery. The study concluded that there is evidence against neurotoxicity in a brief, single lifetime exposure in the childhood period [42]. There have also been several large cohort studies that examine this question but have differing results. The Avon Longitudinal study consisted of 13,433 children and concluded that early childhood anesthesia had no associated long-term neurodegenerative effects. The parameters included general cognitive ability, attention, working memory, and academic performance [43]. However, multiple exposed children were found to have statistically significant decreases in balance, manual dexterity, and social communication scores. The Mayo Anesthesia Safety in Kids study looked at children born between 1996-2000 and found that multiple exposures were associated with an increased risk for learning disabilities and ADHD [44]. A subsequent analysis of 5,339 unexposed and 1,054 singly exposed children from two Olmsted County birth cohorts also found no significant association between single exposures and ADHD, learning capabilities, or a need for personalized education plans [45].

Confounding by indications is currently the main challenge in interpreting observational studies. Children who require a surgical procedure may have underlying chronic conditions or pre-existing developmental vulnerabilities which can increase neurodevelopmental risk. Sophisticated matching and adjustment strategies have significant difficulty in accounting for unmeasured confounding [46]. Most studies have shown consistent associations with multiple exposures and cumulative anesthesia exposures compared to single exposures. However, whether these associations represent causation or confounding by the severity of underlying conditions requiring repeated procedures remains a question.

The current consensus suggests that a brief, single, anesthetic exposure is not associated with long-term deficits in general intelligence. The possibility of effects on behavior, executive function, and social communication remains an area of ongoing research and debate.

## Clinical Implications and Risk Mitigation

**Figure 2:**
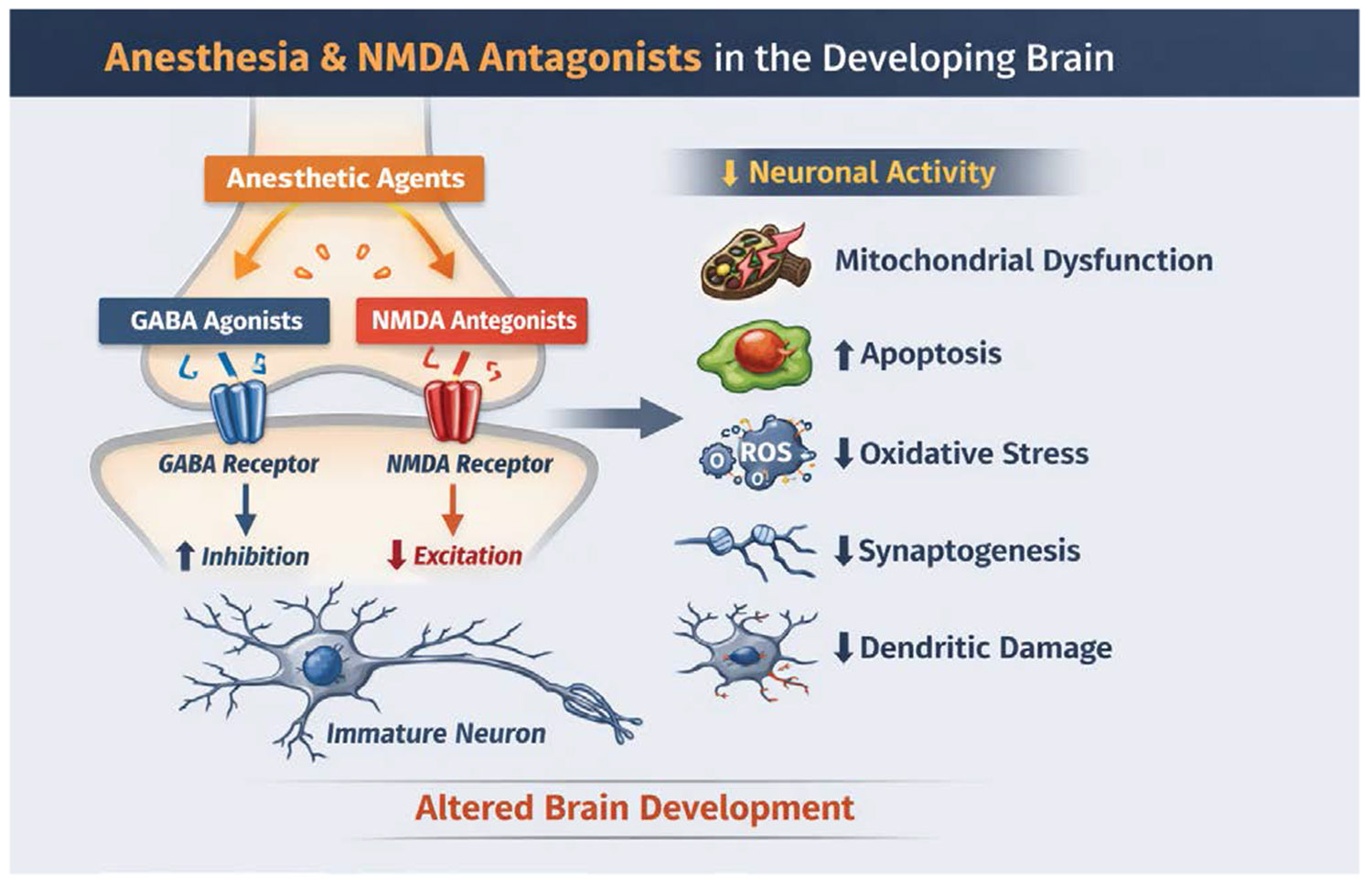
This figure illustrates how anesthetic NMDA receptor antagonists reduce excitatory neurotransmission in the developing brain during critical periods of synaptogenesis. By blocking NMDA receptors, these agents decrease neuronal activity, which can trigger downstream effects including apoptosis, mitochondrial dysfunction, oxidative stress, impaired synaptogenesis, and dendritic structural changes, ultimately contributing to altered neurodevelopment.

There is strong evidence that shows that different anesthetic agents produce distinct neurotoxic effects in the developing brain which have important implications in clinical practice. The γ-aminobutyric acid (GABA) agonists like volatile anesthetics and propofol as well as N-methyl-D-aspartate (NMDA) antagonists like ketamine and nitrous oxide have been shown to have developmental neurotoxicity especially in the early life through various mechanisms [47] (Figure 2).

Volatile anesthetics which bind and potentiate GABA receptors have been widely used as anesthetic agents during procedures. In animal models where neonatal mice were exposed to equivalent doses of isoflurane and sevoflurane, isoflurane showed more neuroinflammation, greater disruption of synaptic proteins and more memory-related protein alterations [48]. Even though isoflurane has shown more drastic effects, a systematic review and meta-analysis of 324 preclinical studies confirmed that sevoflurane, isoflurane, and desflurane all significantly increase neurodegeneration markers like caspase-3, which is an activated cysteine protease playing a vital role in apoptosis [49]. In addition, propofol which works through the NMDA pathway also showed increased caspase-3 levels in the cortex and hippocampus, although research suggests that use of volatile anesthetics like sevoflurane led to higher levels of inflammatory markers [50].

Since volatile anesthetics and propofol have drastic mechanistic differences, this leads to differing long-term effects on the developing brain. Volatile anesthetic exposure during early brain development produces long-lasting effects on GABA receptor mediated inhibitory neurotransmission which can enhance seizure susceptibility in adulthood, whereas ketamine does not lead to the same effects [51]. Recent studies have shown that prolonged neonatal sevoflurane exposure disrupts neuronal maturation and dendritic structure which can result in fine motor ability and spatial memory. The mechanisms through which these anesthetics work play a major role in the lasting effects these agents have on the developing brain [52] (Figure 2).

Apart from the mechanisms, it is important to consider the duration of exposure and frequency to the anesthetic. There was a randomized controlled trial that assigned infants to general anesthesia versus awake-regional anesthesia for inguinal herniorrhaphy, which found no evidence of neurodevelopmental impairment at 5 years of age after a single anesthetic exposure. This leads us to believe that only one small exposure to anesthesia does not significantly alter neurodevelopment in early life [53]. However, research shows that multiple exposures demonstrate a clear association with adverse outcomes. A systematic review and meta-analysis showed statistically significant impairments in children with multiple exposures compared to children with a single exposure. These results were shown through impairments in academics, language and fine motor function. The results also recorded a markedly elevated risk for neurodevelopment disorders and ADHD compared to children with only one exposure [54]. In summary, current research and evidence show that brief, single exposure to anesthetic agents carries minimal detectable risk, while multiple and prolonged periods of exposure have been shown to lead to drastic effects on the developing brain [55].

With evidence showing the neurotoxic effects of anesthetic agents on the developing brain, we must further investigate risk mitigation strategies to try to avoid these detrimental effects. First, we should consider regional anesthetic techniques when feasible, to avoid general anesthesia leading to lower rates of neurotoxic effects. When general anesthesia cannot be avoided, it is important to minimize the exposure duration and unnecessary repeated procedures [56]. Recent studies have shown that using agents like Dexmedetomidine (Precedex), which is an α2-adrenergic agonist, results in less neurotoxic effects and possibly even offers protection against apoptosis, ischemia, and inflammation [57]. In procedural considerations it is important to begin the surgery promptly after induction to minimize the exposure time, while also using appropriate but not excessive anesthetic concentration and ensuring adequate analgesia to prevent any pain-related neurodevelopment effects [58].

## Future Directions and Research Priorities

Despite the tremendous progress in understanding anesthetic neurotoxicity, there are still many questions that remain unanswered, particularly the causality and specific phenotype of injury in people [59]. The most important question that needs to be answered is whether the anesthetic agents cause long-term neurodevelopment effects in children or whether there are confounding variables like surgical conditions, comorbidities or other factors that may play a role in the developing brain. While it is becoming clearer that a single brief exposure to anesthesia in early life is not associated with deficits in broad measures of intelligence, we need to further investigate more specific areas like behavior, executive function, social communication and motor functions [60].

Translation from preclinical to clinical research remains a challenge. Even with vast animal model research which demonstrates neuroapoptosis and behavior changes after exposure to anesthesia, we still have not identified a corresponding phenotype of injury in children [61]. The discordance between preclinical research findings and clinical evidence leads us to more questions about whether we can apply the findings in animal models to human populations, most importantly due to the differences in brain development timing, anesthetic dosing equivalents and the roles of preoperative exposures like pain, inflammation, and surgical stress. When taking these factors into consideration we come closer to bridging the gaps in the research models and preclinical evidence [62].

Another question we must consider is the mechanistic understanding of the developmental neurotoxicity caused by the anesthetic agents. Even with preclinical studies identifying that neuroapoptosis, disrupted synaptogenesis, altered GABAergic neurotransmission, and mitochondrial dysfunction as potential mechanisms for the neurotoxicity, the molecular pathways leading from acute anesthetic exposure to long-term behavioral changes are not as clear [63]. This lack of understanding still leaves a lot of questions unanswered about why anesthesia leads to such detrimental effects.

In addition to the numerous gaps in research that still need to be answered, the need for long term prospective studies into adolescence and adulthood is essential to fully illustrate the neurodevelopmental effects that early life anesthesia exposure leads to. The need for prospective studies would help determine whether early deficits persist, resolve or manifest more detrimental effects later in life [64]. Furthermore, investigating vulnerable populations like children who require multiple anesthesia exposures especially those with already identified developmental disabilities, congenital anomalies, or chronic medical conditions, may be at heightened risk but remain understudied [65].

As discussed previously there have been advances in protective strategies and safer anesthetic protocols. For example, using agents like Dexmedetomidine has emerged as a promising neuroprotective adjuvant which demonstrates less neurotoxicity than traditional anesthetic agents. In addition, Dexmedetomidine has also shown to require reduced opioid requirements, but randomized clinical trials would help solidify these findings [66]. Current research is also investigating novel anesthetic compounds like remimazolam, an ester-analog of midazolam with rapid metabolism, which showed reduced adrenocortical suppression and favorable pharmacological properties. As the research topic focused on new anesthetic agents progresses, this leads to very exciting new possibilities for neuroprotection of the children undergoing anesthetic exposures.

Although significant progress has been made in understanding anesthetic neurotoxicity, critical gaps remain in defining causality, identifying a clear clinical phenotype, and determining long-term outcomes. Brief single exposures appear unlikely to impair global intelligence, yet potential effects on behavior and executive function warrant further study. Translational challenges between animal models and human populations persist, and long-term prospective data especially in vulnerable children are needed, while emerging agents such as dexmedetomidine and remimazolam require further clinical validation.

## Conclusions

Current evidence supports the idea that a single, brief, anesthetic exposure from infancy to adolescence does not result in any long-term cognitive dysfunction. Remaining concerns include prolonged or repeated anesthetic exposures, particularly during windows of synaptogenesis. Animal studies have consistently shown a mechanistic pathway for anesthetic-induced neuronal injury, however, the translation to humans remains unclear and complex which is confounded by surgical pathology, comorbidities, and environmental influences.

Physicians must balance theoretical neurodevelopmental risks against the necessity of timely surgical management, as untreated medical conditions outweigh the harm for potential anesthetic-induced neurotoxicity. Strategies such as minimizing exposure duration, avoiding unnecessary repeat procedures, and regional anesthetic techniques may minimize risk for anesthetic injury in children. These approaches are practical and lower risk and can be implemented while ongoing research is being conducted. Future prospective, longitudinal studies extending from the neonatal period into adolescence and adulthood are crucial for analyzing causality and identifying vulnerable populations to guide safer anesthetic practices. Continued interdisciplinary research will be critical in refining pediatric anesthesia protocols and reassuring families with evidence-based guidance.

## Key Points:

- Early-life exposure to general anesthetics during periods of rapid synaptogenesis has raised concerns about potential neurodevelopmental toxicity in pediatric patients.
- Preclinical studies consistently demonstrate that anesthetic agents can induce neuronal apoptosis, mitochondrial dysfunction, and impaired synaptic development in animal models.
- The primary mechanistic pathways involve NMDA receptor antagonism and GABA receptor potentiation, which suppress neuronal activity during critical developmental windows.
- Additional mechanisms of injury include oxidative stress, epigenetic dysregulation, altered dendritic morphology, and disruption of normal synaptogenesis.
- Clinical studies in humans suggest that a single brief anesthetic exposure in infancy is unlikely to cause measurable deficits in general intelligence or cognitive development.
- Multiple or prolonged exposures to anesthesia in early childhood may be associated with subtle deficits in behavior, executive function, and learning outcomes.
- Strategies to mitigate potential neurotoxicity include minimizing exposure duration, avoiding unnecessary repeated anesthetics, utilizing regional techniques when feasible, and exploring neuroprotective agents such as dexmedetomidine.
- Future research should focus on long-term prospective studies, identifying vulnerable pediatric populations, and developing safer anesthetic agents and protocols.
